# Supplementary material for: Residual physiological abnormalities after pulmonary endarterectomy and balloon pulmonary angioplasty in CTEPH
Source: PLoS One. 2026 Mar 6;21(3):e0344192. doi: 10.1371/journal.pone.0344192 (PMC12965537; doi:10.1371/journal.pone.0344192)
Supplement: S1 Table — (DOCX) [file pone.0344192.s001.docx]

Supplementary Data

# Residual Physiological Abnormalities After Pulmonary Endarterectomy and Balloon Pulmonary Angioplasty in CTEPH

Supplementary Table S1 summarizes patients managed with a hybrid strategy combining pulmonary endarterectomy (PEA) and balloon pulmonary angioplasty (BPA), who were excluded from the main analysis due to heterogeneous treatment pathways.

Three patients underwent PEA before the start of the study and were subsequently treated with BPA after 2017, when the BPA program was implemented at our center. Three additional patients underwent PEA during the study period and received BPA for residual pulmonary hypertension after surgery. One further patient with residual pulmonary hypertension after PEA was referred for BPA but did not complete the program before the study cutoff and was therefore not included.

Not all hybrid patients had complete exercise or quality-of-life data, due to factors like death during follow-up, patient withdrawal, enrollment in a clinical trial for residual pulmonary hypertension, or technical limitations that prevented exercise testing. Among the two patients who did complete exercise right heart catheterization at follow-up, both showed abnormal exercise responses and Physical Component Summary scores below the population norm, despite improvements in resting hemodynamics. Most patients remained on pulmonary vasodilator therapy at follow-up.

| Table S1. Patients Managed with a Hybrid PEA–BPA Strategy | | | | | | | | | | | | | |
| --- | --- | --- | --- | --- | --- | --- | --- | --- | --- | --- | --- | --- | --- |
| Patient ID | Indication for Hybrid strategy type | Year of PEA | Interval PEA–BPA (months) | Baseline mPAP (mmHg) | Baseline PVR (WU) | Follow-up duration  (months) | Last follow-up mPAP (mmHg) | Last follow-up PVR (WU) | mPAP/CO  slope (mmHg/L/min) | PCS (SF-36) | MCS (SF-36) | WHO FC at follow-up | Pulmonary vasodilator therapy |
| H1 | Residual PH | 2011 | 81 | 44.0 | 10.0 | 59 | 38.0^*^ | 6.46^*^ | NA^*^ (enrolled in clinical trial for residual PH) | NA | NA | II | riociguat |
| H2 | Distal disease | 2013 | 49 | 30.0 | 4.9 | 28 | 23.0 | 1.5 | NA (death) | NA | NA | I | riociguat |
| H3 | Residual PH | 2013 | 67 | 39.0 | 11.6 | 49 | 24.0 | 3.9 | 8.1 | 42.5 | 42.2 | I | riociguat |
| H4 | Planned hybrid | 2017 | 9 | 51.0 | 9.8 | 66 | NA^γ^ (withdrawal) | NA^γ^ (withdrawal) | NA^γ^ (withdrawal) | NA | NA | III | bosentano |
| H5 | Residual PH | 2019 | 30 | 32.0 | 7.2 | 33 | 28 | 3.5 | NA (exercise RHC not feasible due to venous access limitations) | 47.3 | 53.2 | I | riociguat |
| H6 | PEA failure | 2021 | 7 | 35.0 | 8.8 | 36 | 22.0 | 1.9 | 5.8 | 35.9 | 59.3 | II | Riociguat, macitentano |

^*^Non-respondent to BPA

^γ^  BPA not completed (only 3 sessions) – the patient refused

NA indicates missing data
