# Supplementary material for: Residual physiological abnormalities after pulmonary endarterectomy and balloon pulmonary angioplasty in CTEPH
Source: PLoS One. 2026 Mar 6;21(3):e0344192. doi: 10.1371/journal.pone.0344192 (PMC12965537; doi:10.1371/journal.pone.0344192)
Supplement: Attachment — Submitted filename: Response to reviewers_II.docx [file pone.0344192.s004.docx]

Reviewer #2: General comments
The revised manuscript is substantially improved compared with the previous version. The tone and conclusions are now better aligned with the descriptive, single-centre observational nature of the dataset. I also appreciate the clearer framing of BPA and PEA as distinct treatment pathways rather than as a head-to-head comparative study, as well as the overall streamlining of the text. The inclusion and description of hybrid cases in the supplementary material is a welcome addition.
Overall, the manuscript is now much closer to being suitable for publication. I have one remaining major point and a small number of minor comments.

Major comment
Operator- and centre-dependence should be more explicitly acknowledged as a determinant of post-interventional improvement
While the Discussion appropriately avoids over-attributing residual abnormalities to fixed “non-curability,” it still under-emphasizes an important practical consideration: both PEA and BPA are highly operator- and centre-dependent interventions. The magnitude of improvement—and the likelihood of residual physiological abnormalities—can vary meaningfully with procedural strategy, completeness of revascularisation, institutional volume, and operator experience/learning curve.

Response:
We thank the reviewer for this important and insightful comment. We agree that operator and center experience represent key determinants of post-interventional outcomes in CTEPH. Accordingly, we have incorporated this consideration explicitly into the Discussion. To support this statement, we added three additional references addressing the learning curve and institutional expertise associated with BPA, as well as the impact of center volume and multidisciplinary experience on outcomes after PEA.

We also revised the Methods section to explicitly describe the institutional and operator experience for both treatment pathways. Specifically, we clarified that PEA procedures were predominantly performed in high-volume referral centers with established expertise in CTEPH management, and that BPA procedures were conducted by the same two dedicated operators, with the initial cases proctored by an experienced high-volume BPA operator. We believe this addition appropriately contextualizes the post-interventional outcomes and acknowledges the potential influence of operator and center experience.

Added references:

37. Brenot P, Jais X, Taniguchi Y, et al. French experience of balloon pulmonary angioplasty for chronic thromboembolic pulmonary hypertension. Eur Respir J 2019;53.

38.	Komura N, Sugano T, Ono F, et al. Effect of balloon pulmonary angioplasty on chronic thromboembolic pulmonary hypertension: an assessment of the learning curve in a Japanese university hospital. Cardiovasc Interv Ther 2025;40:389-

39.	Madani MM, Auger WR, Pretorius V, et al. Pulmonary endarterectomy: recent changes in a single institution's experience of more than 2,700 patients. Ann Thorac Surg 2012;94:97-103.

Minor comments
1. “mean systolic pulmonary arterial pressure” in Figure 4 should be corrected.

Thank you for this observation. The term has been corrected, and the abbreviation “mPAP” is now used in Figure 4 for consistency, as the full term “mean pulmonary arterial pressure” had been previously defined in the manuscript.
